# Antigen-binding affinity and thermostability of chimeric mouse-chicken IgY and mouse-human IgG antibodies with identical variable domains

**DOI:** 10.1038/s41598-019-55805-4

**Published:** 2019-12-17

**Authors:** Juho Choi, Minjae Kim, Joungmin Lee, Youngsil Seo, Yeonkyoung Ham, Jihyun Lee, Jeonghyun Lee, Jin-Kyoo Kim, Myung-Hee Kwon

**Affiliations:** 1 Department of Biomedical Sciences, Graduate School, Ajou University, 206 World Cup-ro, Yeongtong-gu, Suwon, 16499 South Korea; 2 Department of Microbiology, Ajou University School of Medicine, 206 World Cup-ro, Yeongtong-gu, Suwon, 16499 South Korea; 3 Department of Microbiology, Changwon National University, 20 Changwondaehak-ro, Uichang-gu, Changwon, 51140 South Korea

**Keywords:** Molecular biology, Biochemistry, Proteins

## Abstract

Constant (C)-region switching of heavy (H) and/or light (L) chains in antibodies (Abs) can affect their affinity and specificity, as demonstrated using mouse, human, and chimeric mouse-human (MH) Abs. However, the consequences of C-region switching between evolutionarily distinct mammalian and avian Abs remain unknown. To explore C-region switching in mouse-chicken (MC) Abs, we investigated antigen-binding parameters and thermal stability of chimeric MC-6C407 and MC-3D8 IgY Abs compared with parental mouse IgGs and chimeric MH Abs (MH-6C407 IgG and MH-3D8 IgG) bearing identical corresponding variable (V) regions. The two MC-IgYs exhibited differences in antigen-binding parameters and thermal stability from their parental mouse Abs. However, changes were similar to or less than those between chimeric MH Abs and their parental mouse Abs. The results demonstrate that mammalian and avian Abs share compatible V-C region interfaces, which may be conducive for the design and utilization of mammalian-avian chimeric Abs.

## Introduction

Antibodies (Abs) are composed of two identical heavy (H) chains and two identical light (L) chains. The N-terminal domains of both H and L chains, referred to as variable (V) domains, are responsible for antigen (Ag) binding. The rest of the molecules form constant (C) domains. The V and C domains of Abs are structurally and functionally separated; Ag-binding activity is carried out by the V domain, and the C domain is responsible for effector functions. However, a number of recent studies showed that both V and C domains can structurally and functionally influence each other due to mutual cooperation between V (V_H_/V_L_) and C (C_H_/C_L_) domain interfaces. V-to-C domain (V → C) allosteric effects that induce conformational changes in the C domain are transduced by antigen binding^1^, whereas C-to-V domain (C → V) allosteric signals are derived from intrinsic C-domain sequences^2^.

During C → V allosteric signaling, C-domain switching of Abs frequently affects the conformation of the variable (V) region, leading to differences in antigen-binding parameters of V domains, thermodynamics, and functional efficacy^2,3^. This indicates the possibility for affinity modulation through isotype switching without engineering of V domains. These observations were mainly derived from studies on intra-species C-domain switching using V domain-identical murine Abs^4–18^ or V domain-identical human Abs^19–23^. Additionally, some observations arose from studies on inter-species C-domain switching using V domain-identical chimeric mouse-human (MH)-IgGs composed of mouse V_H_ and V_L_ domains with human C_H_ and C_L_ domains^24–28^. However, the consequences of C-domain switching between mammalian and non-mammalian Abs, including avian Abs, remain unknown.

Both humans and mice express Abs with five classes of H chain (μ, δ, γ, ε, and α) and two classes of L chain (κ and λ) comprising different IgM, IgD, IgG, IgE, and IgA isotypes. By contrast, chickens express only three classes of H chain (υ, μ, and α) and a single type of L chain (λ) comprising IgY, IgM, and IgA isotypes. IgY, the major Ab in chickens, is present at high concentrations in serum and egg yolk, and is also transferred from hens to embryos via the egg yolk. IgYs have a molecular mass of ~180 kDa with two H (67–70 kDa each) and two L (25 kDa each) chains that are structurally similar to mammalian IgE comprising four C_H_ domains that lack a hinge region^29^, but they are functionally similar to mammalian IgGs. IgY has two N-glycosylation sites, one in each of the Cυ2 and Cυ3 domains, whereas mammalian IgG has a single N-glycosylation site on the Cγ2 domain, and IgE has seven N-linked glycosylation sites spread across the Cε chain^30^.

Knowledge of how C-domain switching between mammalian and avian Abs affects Ab properties could potentially lead to biotechnology applications for mammalian-avian chimeric Abs. In the present study, we explored C → V allosteric signaling by replacing the C domain (C_γ_ or C_κ_) of mammalian IgG Ab with the corresponding C_υ_ or C_λ_ domain of avian IgY. To investigate how inter-species class switching contributes to Ab properties, we prepared three V domain-identical (parental mouse IgG, chimeric MH-IgG, and chimeric mouse-chicken [MC]-IgY) and two monoclonal (6C407 and 3D8) Abs. We analyzed their antigen-binding parameters and thermodynamic stability using surface plasmon resonance (SPR), intrinsic protein fluorescence, enzyme-linked immunosorbent assay (ELISA), and size-exclusion chromatography (SEC). Both the affinity and thermal stability of MC-6C407 and MC-3D8 IgYs were essentially comparable to those of their respective parental mouse IgGs. By contrast, MH-3D8 IgG differed markedly in terms of thermodynamic stability from its parental mouse 3D8 IgG.

From our results, we concluded that the C domains of chicken Abs are compatible with the V domains of mouse Abs, and can therefore transduce C → V allosteric signals that modulate the features of Abs without destroying them. Our results may assist the design and use of chimeric mammalian-avian Abs. To our knowledge, this is the first study to report the effects of C-region switching between mammalian and avian Abs.

## Results

### Ab production

**Figure 1:**
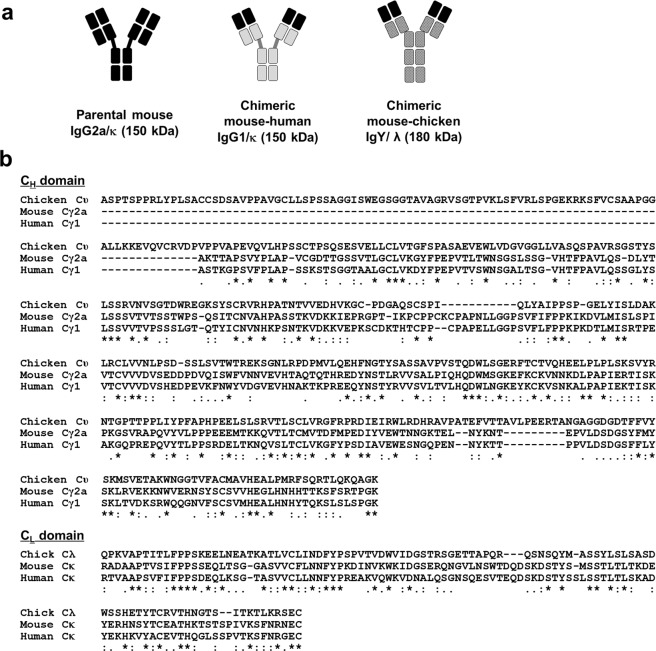
Structure and C-region sequences of Abs. (**a)** Schematic representation of mouse IgG, chimeric MH-IgG (mouse V region with human C regions), and chimeric MC-IgY (mouse V region with chicken C regions). **(b)** Ab sequence alignment. Sequences of constant regions derived from different species were aligned using the Clustal Omega server (https://www.ebi.ac.uk/Tools/msa/clustalo/). Asterisks (*) indicate the positions of single fully conserved residues; colons (:) indicate conservation between groups of highly similar properties (score > 0.5 using the Gonnet PAM 250 matrix); periods (.) indicate conservation between groups of weakly similar properties (score ≤ 0.5 using the Gonnet PAM 250 matrix).

**Figure 2:**
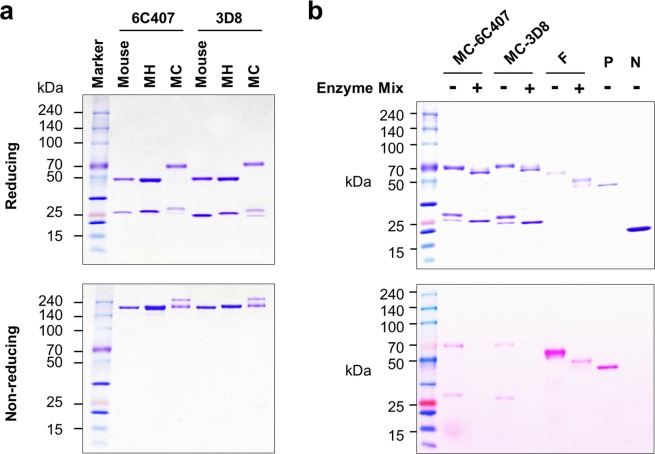
(**a)** SDS-PAGE of purified Abs using a 4–20% gradient polyacrylamide gel. **(b)** Glycosylation analysis. MC-6C407 and MC-3D8 IgY proteins, treated with or without a mixture of glycosylases, were separated by SDS-PAGE followed by Coomassie Blue staining (upper panel) or PAS staining (lower panel). F, fetuin (64 kDa control for deglycosylation conditions); P, horseradish peroxidase (44 kDa positive control for glycoprotein staining); N, soybean trypsin inhibitor (20 kDa negative control for glycoprotein staining).

Herein, we prepared three anti-KIFC1 Abs (6C407) and three anti-nucleic acid Abs (3D8). Each panel includes V region-identical mouse IgG2a/κ, chimeric MH-IgG1/κ, and chimeric MC-IgY/λ Ab types. The predicted structures of the expressed proteins are shown in Fig. 1a. Amino acid sequences for the C regions of mouse IgG2a/κ, human IgG1/κ, and chicken IgY/λ are shown in Fig. 1b. Two original mouse Abs, 6C407 IgG2a/κ and 3D8 IgG2a/κ, were purified from the culture supernatant of their respective hybridoma cells using Protein L-agarose resin. The two chimeric MH-IgGs were purified from the culture supernatant of FreeStyle 293-F cells transfected with the specific KV10 vectors using Protein A-agarose resin at 7 days post-transfection, and the two chimeric MC-IgYs were purified using Protein L-agarose. SDS-PAGE analysis showed that mouse IgGs (6C407 and 3D8) and MH-IgGs (6C407 and 3D8) were of the expected sizes (~50 and ~25 kDa, respectively) under reducing conditions and >150 kDa under non-reducing conditions (Fig. 2a). Interestingly, MC-IgYs (6C407 and 3D8) yielded a single band of ~70 kDa for the υ H chain, and two distinct bands (~25 and ~27–28 kDa) for the λ L chain under reducing conditions. MC-IgYs also yielded two distinct bands (>150 and >200 kDa) under non-reducing conditions. This two-band appearance for IgY molecules has not been reported previously.

Next, we investigated whether chimeric MC-IgY proteins expressed in FreeStyle 293-F cells were glycosylated, based on the assumption that differences in glycosylation state may explain the two bands running at ~25 kDa under reducing conditions and the presence of two bands under non-reducing (native) PAGE. Coomassie Blue staining yielded protein bands with a slightly lower molecular mass following reaction with the deglycosylation enzyme cocktail (Fig. 2b, upper panel). In parallel, the fetuin positive control for deglycosylation shifted from 64 to 55 kDa following deglycosylation enzyme cocktail treatment. Signals corresponding to the H and L chains of MC-IgYs were not detected by PAS staining after reaction with the deglycosylation enzyme cocktail, whereas a signal was detected with unreacted MC-IgYs (Fig. 2b, lower panel). This indicates that both υ H and λ L chains in MC-IgYs were glycosylated. Thus, the presence of two bands in MC-IgYs could be explained by partial glycosylation of both Cυ H and Cλ L chains. This is in contrast with previous reports for natural IgY Abs purified from chicken serum in which IgYs contain two potential N-glycosylation sites located in Cυ2 and Cυ3 domains^30^.

### Class switching to chicken C regions affects antigen-binding parameters

**Figure 3:**
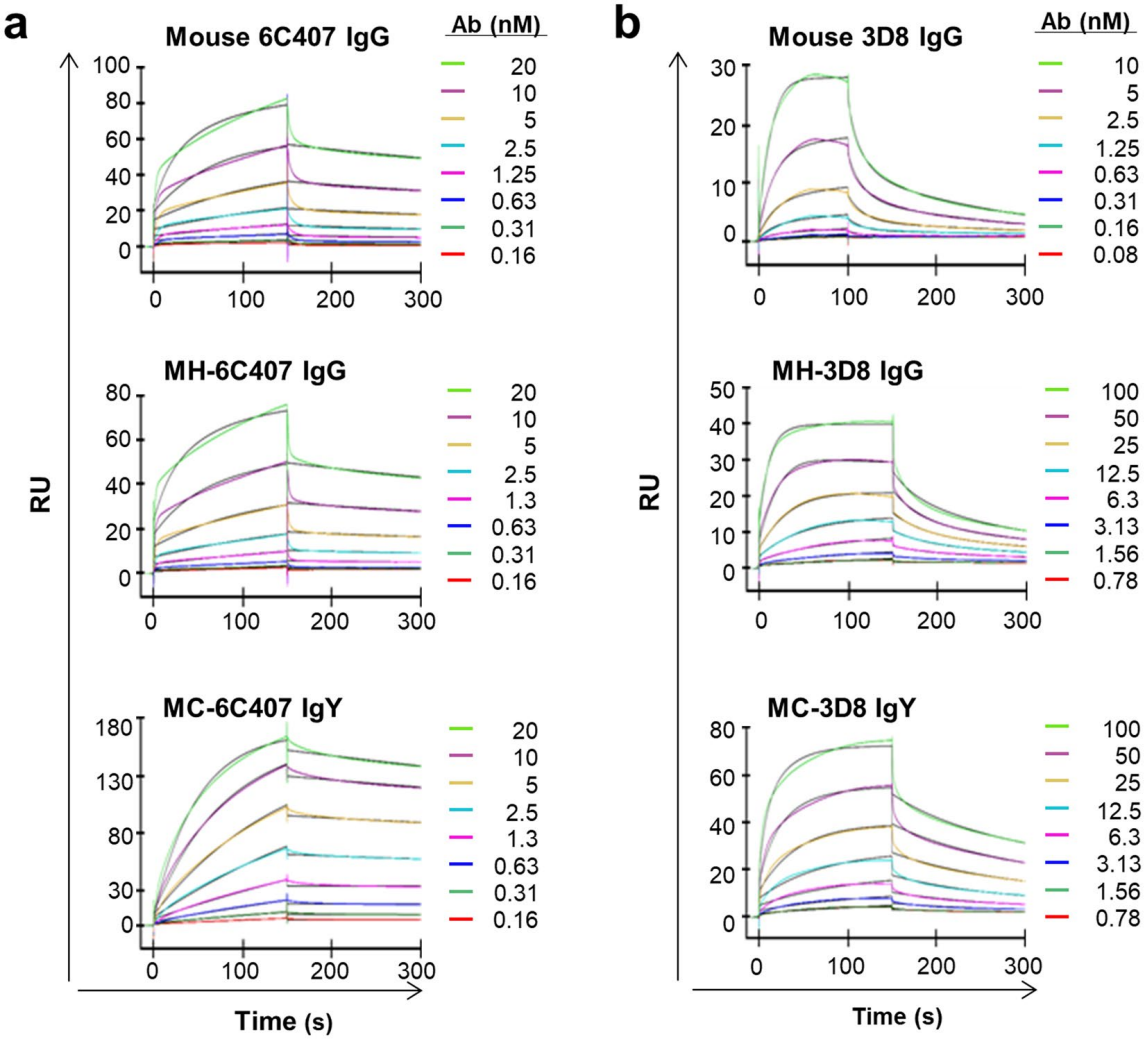
SPR analysis of the kinetics of Ab-antigen interactions. SPR sensorgrams are shown for 6C407 binding to the 12 amino acid peptide antigen **(a)** and 3D8 binding to the ss-(dN)_40_ oligonucleotide antigen **(b)**. At least five concentrations of each Ab were injected over the biotinylated antigen immobilized on a *streptavidin* sensor chip. Experimental data were plotted together with curves drawn from a fitted 1:2 Langmuir isotherm. Colored and black lines indicate recorded and calculated curves, respectively. RU, resonance unit.

Next, we measured binding parameters (*k*_on_, *k*_off_, and *K*_D_) for the three-member panels of 6C407 and 3D8 Abs by SPR (Fig. 3 and Table 1). The binding affinity of MH-6C407 was almost the same as that of parental mouse 6C407, whereas MC-6C407 displayed ~2-fold higher affinity than parental mouse 6C407. In the case of 3D8 Abs, the binding affinity of chimeric MC-3D8 was almost the same as that of parental mouse 3D8, while that of MH-3D8 was ~2-fold lower than that of parental mouse 3D8. These results indicate that mouse Abs can tolerate the replacement of their C regions (C_γ_ and C_κ_) by the corresponding avian C regions (C_υ_ and C_λ_) without destroying activity, consistent with the tolerance to switching of C regions between mammalian Abs, although antigen-binding kinetics are affected to some extent.

**Table 1:** Binding kinetics and affinity^a^ of Ig proteins for their antigens^b^.

Ab	Type	*k*_on_ (M^−1^ s^−1^)	*k*_off_ (s^−1^)	*K*_D_ (M)
6C407	Mouse IgG2a/κ	(5.67 ± 0.11) × 10^5^	(1.38 ± 0.04) × 10^−3^	2.45 × 10^−9^
	Chimeric MH (IgG1/κ)	(5.82 ± 0.07) × 10^5^	(1.31 ± 0.02) × 10^−3^	2.25 × 10^−9^
	Chimeric MC (IgY/λ)	(5.11 ± 0.01) × 10^5^	(6.32 ± 0.03) × 10^−4^	1.24 × 10^−9^
3D8	Mouse IgG2a/κ	(6.04 ± 0.15) × 10^6^	(1.36 ± 0.06) × 10^−1^	2.25 × 10^−8^
	Chimeric MH (IgG1/κ)	(5.08 ± 0.07) × 10^5^	(2.42 ± 0.03) × 10^−2^	4.77 × 10^−8^
	Chimeric MC (IgY/λ)	(2.72 ± 0.02) × 10^5^	(7.00 ± 0.08) × 10^−3^	2.56 × 10^−8^

^a^The K_D_ values were calculated by analyzing at least five data sets using different protein concentrations.

^b^The 12-amino acid length peptide antigen labeled with biotin at the N-terminal for 6C407. The single-stranded oligodeoxynucleotide labeled with biotin at the 5′ end [bio-ss-(dN)_40_] for 3D8. Each value represents the mean ± SD of two independent experiments.

### Structural stability of Abs under thermal stress

**Figure 4:**
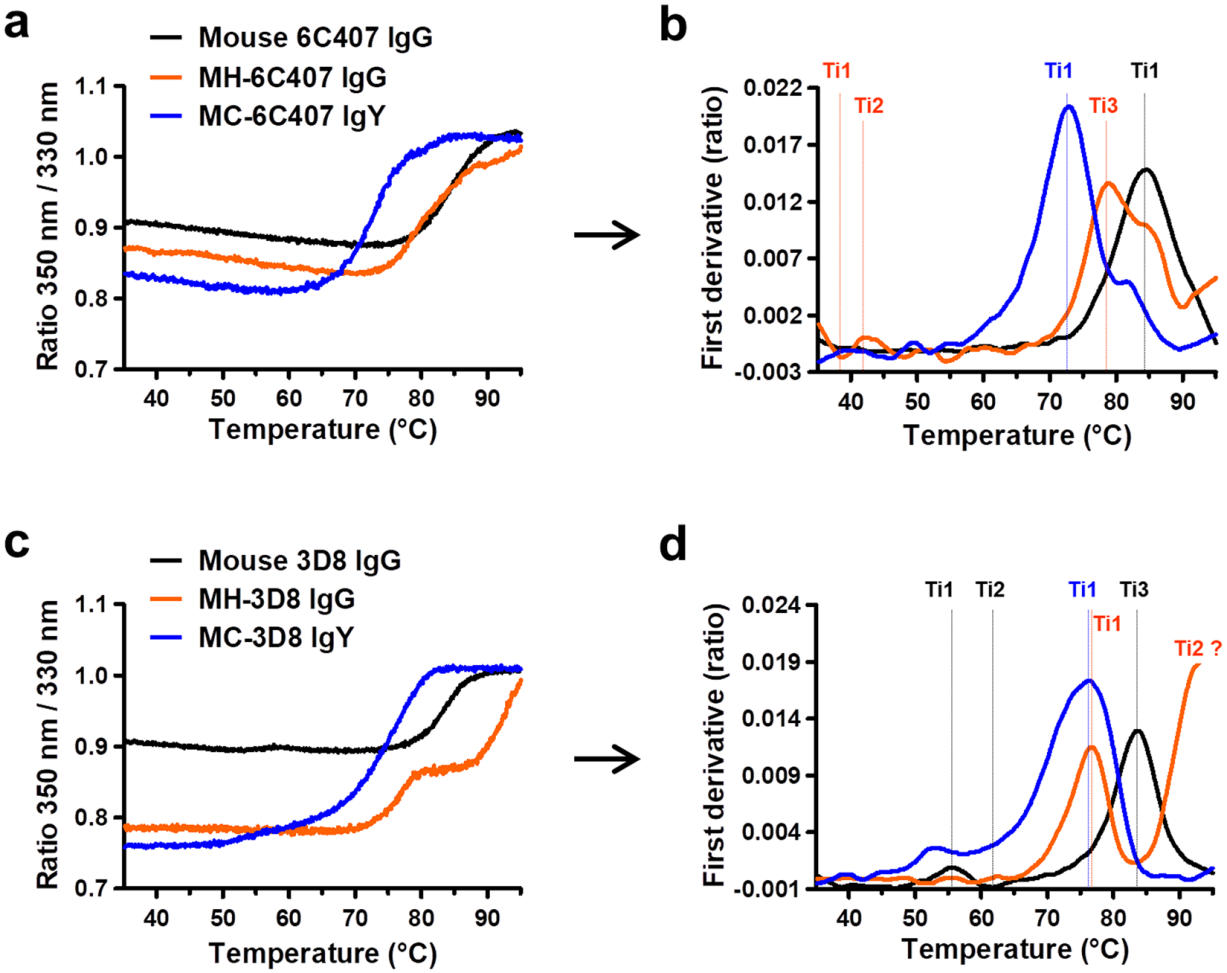
Analysis of Ab stability based on thermal unfolding curves. **(a**,**c)** F350/F330 fluorescence ratio. **(b**,**d)** Corresponding thermal shifts (first derivative profiles). Values represent averages with standard deviation from triplicate measurements.

We compared the structural stability of the six Abs (three 6C407 and three 3D8 Abs) under thermal stress by analyzing intrinsic protein fluorescence using a Tycho NT.6 system. Parental mouse 6C407 and MC-6C407 yielded a single inflection temperature (Ti) value that is the temperature at which an unfolding transition occurs, calculated from the F350/F330 ratio of the fluorescence intensity at 350 vs. 330 nm, where tryptophan and tyrosine fluoresce in the unfolded and folded states, respectively. By contrast, three Ti values (38.6 °C, 42.1 °C, and 78.8 °C) were detected for MH-6C407, indicating three unfolding events (Fig. 4a,b). The major Ti values for the three 6C407 Abs were 84.6 °C (Ti 1), 78.8 °C (Ti 3), and 72.9 °C (Ti 1) for mouse 6C407, MH-6C407, and MC-6C407, respectively (Fig. 4a,b and Table 2). Thus, compared with mouse 6C407, both MH- and MC-6C407 underwent a leftward shift in Ti value to lower temperature (Fig. 4a,b), and this shift to lower temperature was more pronounced in MC-6C407 than MH-6C407.

**Table 2:** Summary of the thermal shift analysis.

Ab	Type	Ti 1 (°C)	Ti 2 (°C)	Ti 3 (°C)	Initial Ratio	ΔRatio
6C407	Mouse IgG2a/κ	84.6			0.9088	0.1270
	Chimeric MH (IgG1/κ)	38.6	42.1	78.8	0.8704	0.1473
	Chimeric MC (IgY/λ)	72.9			0.8346	0.1892
3D8	Mouse IgG2a/κ	83.7	61.7	83.7	0.9064	0.1031
	Chimeric MH (IgG1/κ)	76.7	>90 °C?		0.7861	0.2190
	Chimeric MC (IgY/λ)	76.3			0.7605	0.2487

Initial ratio: the 350 nm/330 nm fluorescence ratio at the start of the experiment (at 35 °C). ∆Ratio: difference between the ratio at the beginning and at the end of the thermal profile. Ti, the inflection temperature at which an unfolding transition occurred.

In the 3D8 three-member panel, three Ti values were detected for parental mouse 3D8, compared with a single Ti value for the two chimeric 6C407 Abs. A leftward shift in major Ti value to lower temperature was detected for MH-3D8 (76.7 °C) and MC-3D8 (76.3 °C), compared with parental mouse 3D8 (83.7 °C), indicating a decrease in thermal stability for these Abs (Fig. 4c,d and Table 2). Interestingly, MH-3D8 displayed a dramatic increase in F350/F330 ratio at 85 °C, indicating the existence of a domain that retains structural stability over 90 °C, but this temperature could not be achieved using this measurement system. Taken together, the results suggest that replacement of the mouse C domain with a chicken C domain causes a decrease in thermal stability to a slightly greater extent than C-region switching between mouse and human Abs.

The change in F350/F330 ratio differed at the beginning and end of the thermal profile, and was greatest for the two chimeric MC Abs (0.1892 and 0.2487), followed by the two chimeric MH Abs (0.1473 and 0.2190), and the parental mouse Abs (0.1270 and 0.1031), as shown in Table 2. Accordingly, the structures of MC-chimeric Abs appear to be more susceptible to thermal stress than those of IgGs.

### Functional stability and aggregation/degradation behavior of Abs under thermal stress

**Figure 5:**
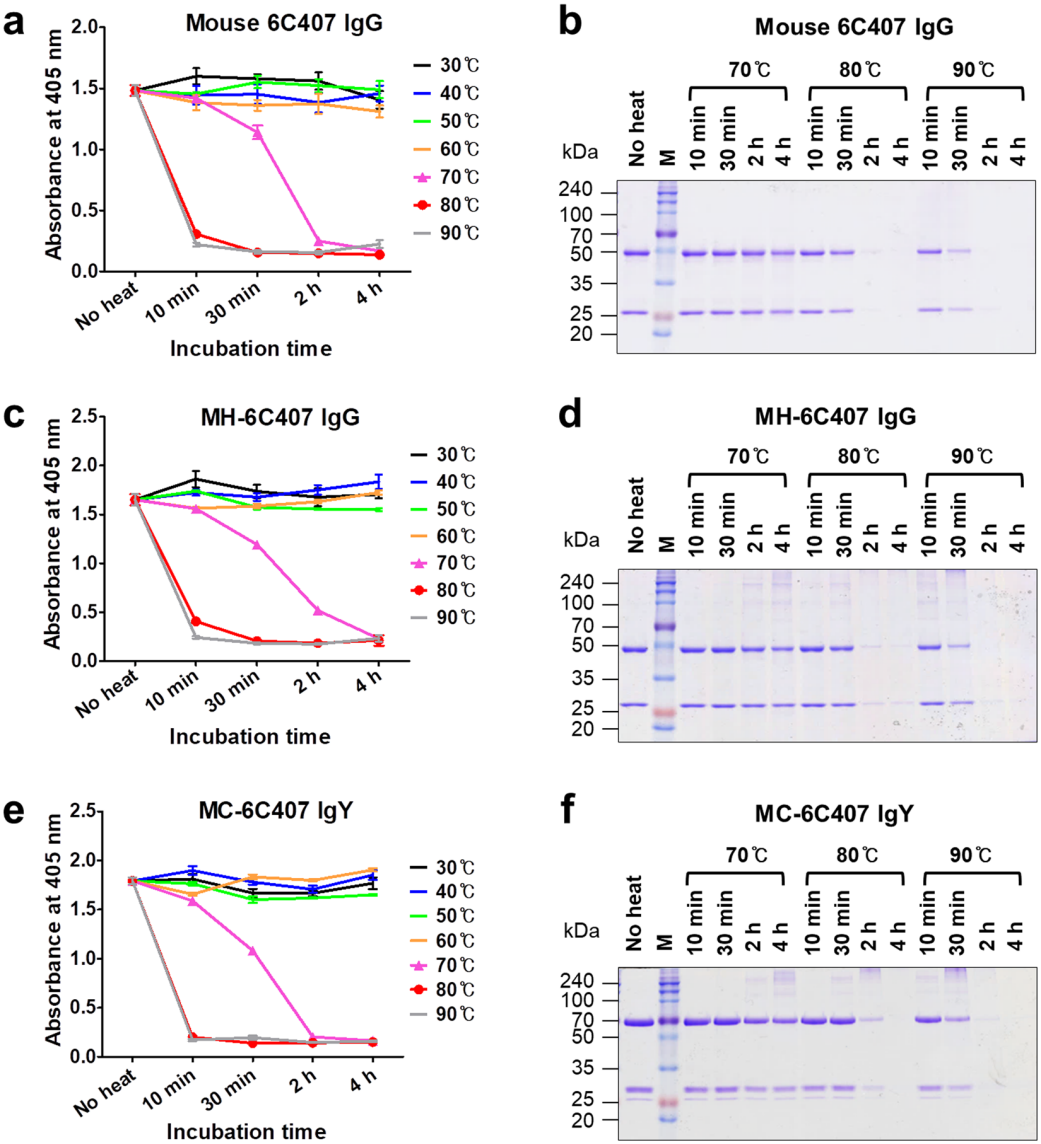
ELISA and SDS-PAGE analysis under thermal stress. (**a**,**c**,**e)** ELISA of the antigen-binding activity of 6C407-derived antibodies. Purified 6C407 antibodies were heated under the specified conditions, placed in wells coated with KIFC1_43–54_ peptide (EDGLEPEKKRTR), and bound 6C407 antibodies were detected with AP-conjugated antibodies specific for mouse IgG/Fc (**a**), human IgG/Fc (**b**), or chicken IgY/υ chain (**c**). Data are presented as mean ± SD (n = 3). **(b**,**d**,**f)** SDS-PAGE analysis of antibody integrity. Purified 6C407 antibodies were heated under the specified conditions then subjected to SDS-PAGE under reducing conditions using a 12% polyacrylamide gel, followed by staining with Coomassie Blue.

We analyzed the antigen-binding activity and aggregation/degradation behavior by ELISA and SDS-PAGE, respectively, after heating Abs for 10 min to 4 h at 70–90 °C. The three 6C407 Abs displayed comparable functional stability, and all retained antigen-binding activity after heating at 60 °C for 4 h and at 70 °C for 10 min (Fig. 5). A loss in antigen-binding activity was first observed after heating at 70 °C for 30 min, and thermal stress at 80 °C for 10 min caused the loss of more than ~90% of activity (Fig. 5a,c,e).

Noncovalent aggregation and degradation behavior analyzed by reducing SDS-PAGE was similar among the three types of Abs, with all undergoing dramatic aggregation following heating at 80 °C and 90 °C for 2 h (Fig. 5b,d,f). Protein bands above 50 kDa, observed with increasing incubation time and temperature, are thought to be temporary aggregation intermediates undergoing protein degradation^31^.

**Figure 6:**
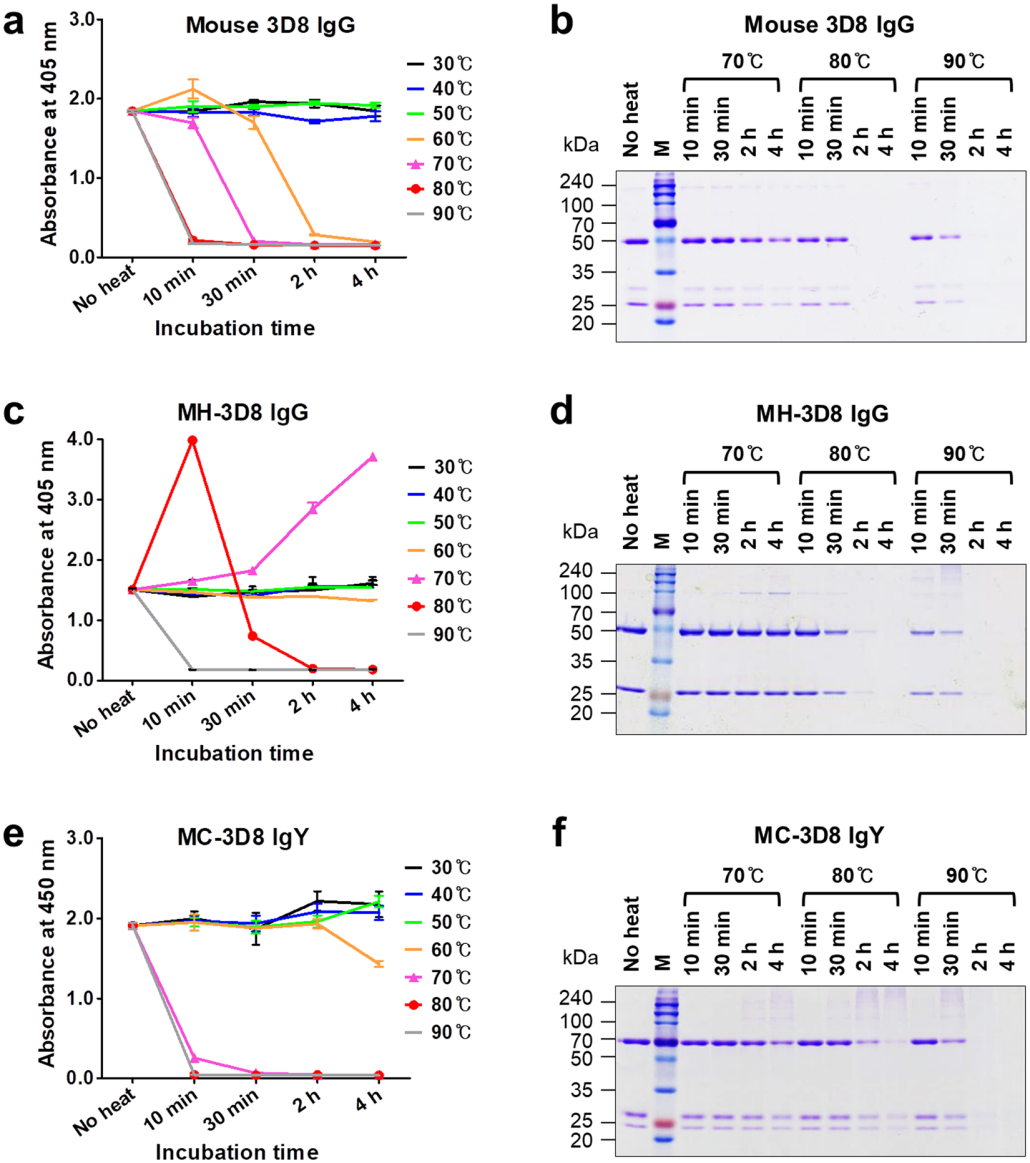
ELISA and SDS-PAGE analysis under thermal stress. (**a**,**c**,**e)** ELISA of the antigen-binding activity of 3D8 antibodies. Purified 3D8-derived antibodies were heated under the specified conditions, placed in wells coated with plasmid DNA antigen (pUC19), and bound 3D8 antibodies were detected with AP-conjugated antibodies specific for mouse IgG/Fc (**a**), human IgG/Fc (**b**), or chicken IgY/υ chain (**c**). Data are presented as mean ± SD (n = 3). **(b**,**d**,**f**) SDS-PAGE analysis of antibody integrity. Purified 3D8 antibodies were heated under the specified conditions then subjected to SDS-PAGE under reducing conditions using a 12% polyacrylamide gel, followed by staining with Coomassie Blue.

**Figure 7:**
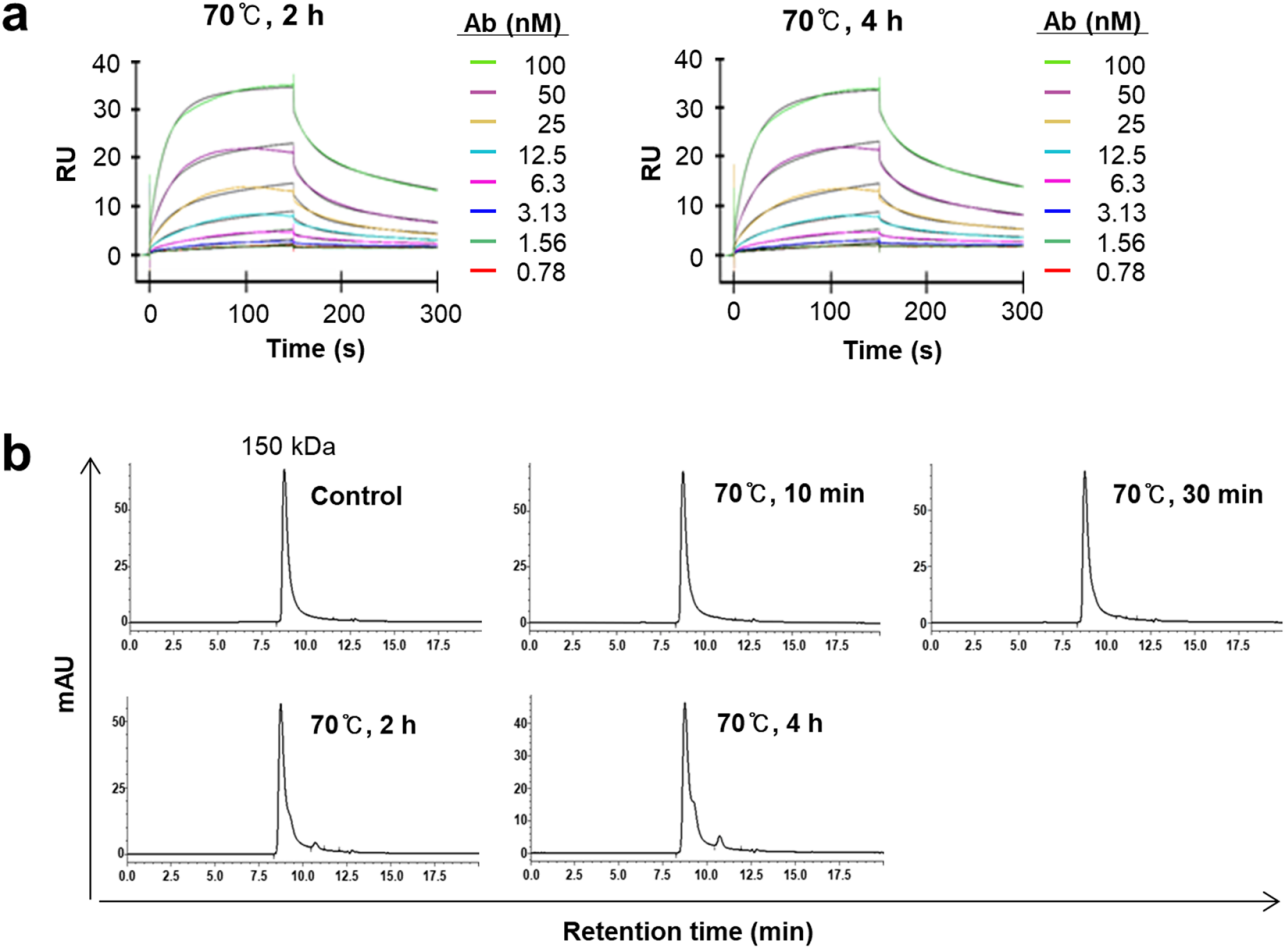
Analysis of MH-3D8 heated at 70 °C for 2 or 4 h. (**a)** SPR analysis of kinetics using the ss-(dN)_40_ oligonucleotide antigen. Colored and black lines indicate recorded and calculated curves, respectively. RU, resonance unit. **(b)** SEC analysis. The indicated molecular weight was interpolated using a standard curve generated with proteins of known mass and retention time.

The three types of 3D8 Abs displayed distinct differences in functional stability (Fig. 6). Parental mouse 3D8 lost ~90% of its antigen-binding activity after incubation at 60 °C for 2 h, whereas MC-3D8 retained activity after incubation at 60 °C for 2 h but lost ~90% of the activity after harsher thermal stress treatment at 70 °C for 10 min (Fig. 6a,e). Protein bands corresponding to mouse 3D8 IgG, MH-3D8, and MC-3D8 disappeared following thermal stress at 80 °C and 90 °C for 2 h (Fig. 6b,d,f). Interestingly, the activity of MH-3D8 was increased following thermal stress at 70 °C for 2 h, 70 °C for 4 h, and 80 °C for 10 min (Fig. 6c). However, inconsistently, the affinity of these heat-treated MH-3D8 molecules measured by SPR was ~10-fold lower than that of untreated MH-3D8 (Fig. 7a and Table 3). This discrepancy may be caused by differences in experimental procedures; an anti-Fc Ab was used in ELISA but not in SPR experiments. In ELISA, the Fc structure may be altered within a specific window of thermal stress, and this may be better recognized by an anti-Fc Ab. The SEC profile showed single peaks corresponding to an apparent 150 kDa IgG (Fig. 7b), although slight aggregation was observed for heat-treated MH-3D8 in SDS-PAGE (Fig. 6d), indicating no aggregation or degradation. Therefore, it seems that a subtle change in MH-3D8 structure, rather than aggregation, is responsible for the enhanced DNA-binding activity within a specific window of thermal stress. This conclusion is supported by the emergence of an additional unfolding transition only for MH-3D8 at temperatures >90 °C (Fig. 4d). An Ab region corresponding to this shift in unfolding transition may be the Fc region that is not responsible for DNA binding.

**Table 3:** Binding kinetics and affinity^a^ of Ig proteins for their antigens^b^.

Ab	Heating	*k*_on_ (M^−1^ s^−1^)	*k*_off_ (s^−1^)	*K*_D_ (M)
Chimeric MH-3D8	No	(5.08 ± 0.07) × 10^5^	(2.42 ± 0.03) × 10^−2^	4.77 × 10^−8^
	70 °C, 2 h	(1.30 ± 0.01) × 10^5^	(4.00 ± 0.03) × 10^−2^	3.05 × 10^−7^
	70 °C, 4 h	(1.16 ± 0.01) × 10^5^	(4.00 ± 0.03) × 10^−2^	3.44 × 10^−7^

^a^The K_D_ values were calculated by analyzing at least five data sets using different protein concentrations.

^b^The single-stranded oligodeoxynucleotide labeled with biotin at the 5′ end [bio-ss-(dN)_40_].

## Discussion

In this study, we demonstrate that chimeric MC-IgY Abs allow modulation of antigen-binding parameters and thermal stability, as has been described for chimeric MH-IgGs. Both V and C domains are responsible for Ab structure and function, as demonstrated recently by analyzing different mammalian (mouse, human, and chimeric MH) Abs with identical V regions. The current study further supports this notion through C-region switching between mammalian and avian Abs with identical V regions. This study also expands our understanding of C-region switching between Abs of evolutionarily distant species, and the findings may have implications for improving Ab engineering, and thus possible utilization of Abs in biotechnology.

Most previous studies on the effects of class switching have focused on C_H_ switching of Abs within a species, while C_L_ regions are not switched. V region-identical mouse IgG1, IgG2a, IgG2b, and IgG3 Ab subclasses exhibited differences in affinity and thermodynamics. Moreover, the affinity and thermodynamics between IgG2a and its Fab form (IgG2a-Fab), as well as between IgG3 and its Fab form (IgG3-Fab), are known to differ^10^. Structural differences imposed by the C_H_ chain of Abs have been proven using small-angle X-ray scattering measurements^14^. V region-identical human IgA1/κ, IgG1/κ, and even Fab fragments derived from these Abs exhibit significantly different affinities^23^. Thermodynamic analysis of the binding of IgGs and their Fabs showed that Fc regions contribute strongly to affinity by inducing conformational changes in V regions through an allosteric mechanism^32^. Even mutation at two amino acids in IgA Fc (C_H_α3 domain) resulted in a ~20-fold reduction in antigen-binding affinity compared with that of wild-type IgA^33^. These studies demonstrate that conformational changes in the V region can be dictated by C_H_ domains (such as hinge and Fc regions), and are not limited by the C_H_1 domain. On the other hand, studies focusing on C_L_ switching were performed using the Fab format rather than the full-size Ig format^34,35^. C_κ_-to-C_λ_ chain switching in catalytic Abs can alter structure and function^34^, and the thermodynamic stability of human Fabs can differ depending on the type of C_L_ chain (C_κ_ or C_λ_) present in the Ab^35^. Class switching and the structural effects of V regions can differ depending on which class of C_H_ or C_L_ is present, and whether full-size IgG or Fab forms are studied. Since the chimeric MC-IgY Abs in the present study were produced by replacing both mouse C_γ_ and C_κ_ domains with chicken C_υ_ and C_λ_ domains, changes in the antigen-binding parameters and thermal stability of MC-IgY Abs are likely due to the properties of C_υ_ and C_λ_ chains that mediate an intrinsic C → V allosteric signal.

Two chimeric MC Abs (MC-6C407 and MC-3D8 IgYs) displayed only subtle differences in antigen-binding kinetics and thermal stability, compared with parental mouse Abs. This suggests that the C regions (C_υ_ and C_λ_) of chicken IgYs are compatible with the V regions of mouse Abs, even though birds and mammals diverged from their common ancestor >300 million years ago^36^. This compatibility of V-C region interfaces between mouse and chicken Abs is supported by a report showing that chimeric chicken-human Abs composed of chicken V domains and human C domains exhibit similar binding affinity to parental chicken Abs^37^. Furthermore, V-C region compatibility between these species has been observed in a chickenized single-chain variable fragment (scFv) Ab harboring complementarity-determining regions (CDRs) of the mouse Ab and framework regions (FRs) of a chicken Ab^38^.

IgYs do not show cross-reactivity to mammalian IgGs, i.e., Abs raised against IgYs do not bind to mammalian IgGs due to immunological differences between IgYs and mammalian IgGs. Also, the C region of IgYs does not bind to mammalian Fc receptors. This feature makes chicken IgY Abs particularly valuable in experimental research and in immunodiagnostic assays, such as immunohistochemistry, Western blotting, flow cytometry, and ELISA, all of which require low signal-to-noise ratios. These properties of IgYs, which mainly stem from the C region of IgY, could also be shared by chimeric MC-IgYs. The current results suggest that Ab engineering using the C region of chicken IgYs may expand the biotechnological applications of chimeric MC-IgYs. For example, indirect sandwich ELISA usually requires two Abs derived from different species that recognize different epitopes, and one of two Abs can be obtained by replacing the C region of a mammalian IgG with the C region of a chicken IgY following the production of two monoclonal IgG Abs from a single mammalian host. In other words, construction of chimeric MC-IgYs from mammalian IgGs could eliminate the inconvenience of using antibodies from different mammalian species in laboratory assays.

The thermodynamic stability of Abs is associated with the subclass of human IgGs rather than the V domains^39^. However, all three V region-identical types of 6C407 Abs (mouse 6C407, MH-6C407, and MC-6C407) were similar in terms of thermal stability evaluated by antigen-binding activity (Fig. 5), but there were remarkable differences between the three V region-identical types of 3D8 Abs (Fig. 6). This suggests that both V and C domains of Abs can influence their thermal stability. Interestingly, analysis of thermal stability of Abs based on antigen-binding activity was not consistent with analysis based on monitoring fluorescence. The fluorometric method indicated that the conformational structure of the two chimeric MC-IgYs (MC-6C407 and MC-3D8) was more susceptible to thermal stress than that of their corresponding parental mouse IgGs and MH-IgGs (Fig. 4 and Table 2), demonstrating that MC-IgYs are thermodynamically less stable. This tendency has been reported in a previous study in which the thermal stability of a chicken IgY measured by antigen-binding activity was similar to that of cow, goat, and pig IgGs, whereas the conformational stability of the chicken IgY measured using a fluorometric method was lower^40^.

Only correctly folded and assembled Abs that pass through the endoplasmic reticulum (ER) quality-control system are secreted from Ab-producing cells^41^. Under the ER quality-control system, typical IgGs assemble first as H chain dimers that are incompletely folded and associated with the molecular chaperone of binding immunoglobulin protein (BiP) in the ER until they associate with cognate chains. Complete folding of C_H_1 occurs only after interaction with the folded C_L_ domain^42,43^. The ER quality-control machinery, first identified in B lineage cells, is now known to function in Chinese hamster ovary (CHO) cells used for therapeutic Ab production^44^. Recently, however, it was found that this machinery does not work on some engineered IgG Abs such as humanized IgGs, as evidenced by secretion of dimeric H chain-only IgGs not associated with L chains in CHO cells^45^. Thus, evading this machinery may be dependent on the intrinsic properties of the V_H_ domain because engineered V_H_ domains of IgGs confer complete folding on C_H_1 domains to evade ER retention^45^. We predict that this machinery may also function in FreeStyle 293-F cells used for Ab production in the present work. Chimeric MH-IgG and MC-IgY Abs were efficiently secreted in an assembled form from FreeStyle 293-F cells (Fig. 2a), yielding ~5 mg antibody per 100 ml cell culture. Therefore, these chimeric Abs might pass the ER quality-control system, suggesting that mouse V_H_ domains confer incomplete folding on chicken C_υ_1 and human C_γ_1 domains until they are associated with chicken C_λ_ and human C_κ_ domains, respectively, in the ER.

## Materials and Methods

### Construction of recombinant Abs

Genes encoding V regions of two mouse monoclonal Abs (3D8 and 6C407) were cloned into KV10 plasmids for expression of chimeric Abs (3D8 V_H_, GenBank accession number AAF79128; 3D8 V_L_, AAF79129; 6C407 V_H_, MH638366; 6C407 V_L_, MH638367). 3D8 and 6C407 Abs are specific for DNA and KIFC1 antigens, respectively. The KV10H vector contains human C_γ_1, C_γ_2, C_γ_3, and C_κ_ genes under the control of two individual cytomegalovirus (CMV) promoters (P_CMV_) that allow simultaneous expression of H and L chains. The KV10C vector contains chicken C_υ_1, C_υ_2, C_υ_3, C_υ_4, and C_λ_ genes. The KV10 series are built around a cassette vector that permits the cloning of all types of Ig H and L chains with leader sequences upstream using specific restriction enzymes, and facilitates individual cloning of Ab fragment gene cassettes (V and C domains) into specific cloning sites. Genes encoding V_H_ and C_H_ chains were flanked with *Mfe*I/*Nhe*I and *Nhe*I/*BamH*I restriction enzyme sites, respectively, while V_L_ and C_L_ genes were flanked with *Bgl*II/*BsiW*I and *BsiW*I/*EcoR*I sites. Genes encoding V_H_ regions of Abs were cloned upstream of C_H_1 using *Mlu*I/*Nhe*I restriction sites, and V_L_ genes of Abs were cloned upstream of the respective C_L_ using *Dra*III/*BsiW*I restriction sites.

### Preparation of Ab proteins

To prepare mouse Ab proteins, mouse hybridoma cells were cultured in RPMI1640 media (ThermoFisher Scientific; cat# 11875-093) supplemented with 10% fetal bovine serum (FBS) at 37 °C in an atmosphere of 5% CO_2_. Mouse 3D8 IgG2a/κ and 6C407 IgG2a/κ were purified from the supernatant of hybridoma cells, and 3D8 IgG and 6C407 IgG were purified by affinity chromatography using Protein L-agarose resin that binds to the Vκ region of Abs (GE Healthcare; cat# 17-5478-01). Chimeric Ab proteins were prepared from cultures of FreeStyle 293-F serum-free and suspension-adapted HEK293F cells (ThermoFisher Scientific; cat# R79007). HEK293F cells were cultured in serum-free FreeStyle 293 media (ThermoFisher Scientific; cat# 12338018) with 8% CO_2_ and shaking at 130 rpm in the 37 °C incubator. FreeStyle 293-F cells (100 ml) at a density of 2 × 10^6^ cells/ml were transfected with 200 μg of KV10 plasmid encoding an Ab gene using 400 μg of polyethylenimine (PEI) reagent with a molecular weight of ~25 kDa (Polyscience; cat# 23966-2), a final PEI concentration of 4 μg/ml^46^. After 7 days, the culture supernatants were harvested by centrifugation, and two chimeric MC-IgY and two chimeric MH-IgG proteins were purified by affinity chromatography using Protein L (GE Healthcare; cat# 17-5478-01) and Protein A column (GE Healthcare; cat# 17-1279-01), respectively.

### Deglycosylation of chimeric MC-IgYs

Chimeric MC-IgY proteins (5 μg) were incubated with a deglycosylation enzyme mixture (New England Biolabs) containing O-glycosidase, PNGase F, neuraminidase (sialidase), β1–4 galactosidase, and β-N-acetylglucosaminidase for 4 h at 37 °C according to the manufacturer’s guidelines (New England Biolabs; cat# P6039S).

### Periodic acid-Schiff (PAS) staining of chimeric MC-IgYs

Chimeric MC-IgY proteins (5 μg), before and after reaction with a deglycosylation enzyme cocktail, were subjected to sodium dodecyl sulfate-polyacrylamide gel electrophoresis (SDS-PAGE), and PAS staining was performed to detect protein-bound carbohydrates using a glycoprotein staining kit according to the manufacturer’s instructions (ThermoFisher Scientific; cat# 24562).

### Surface plasmon resonance (SPR)

Measurement of binding parameters between Abs and their antigens was performed using a BIAcore T200 instrument (GE Healthcare Life Science) at 25 °C. Abs were diluted in HBS-EP (10 mM HEPES pH 7.4, 250 mM NaCl, 3 mM EDTA) containing 0.05% (v/v) surfactant P-20. The same buffer was used as the running buffer. For both 6C407 anti-KIFC1 and 3D8 anti-DNA Ab series, a 12 amino acid peptide (HSET-N) labeled with biotin at the N-terminus (bio-EDGLEPEKKRTR) and a single-stranded (ss) oligonucleotide (ss-(dN)_40_) labeled with biotin at the 5′-end (5′-bio-CCATGAGTGATAACACTGCGGCCAACTTACTTCTGACAAC-3′) were respectively immobilized on a Series S sensor chip SA (GE Healthcare; cat# 29-1049-92) at a level of 10–40 response units. Diluted Ab proteins were injected into the flow cell for 3 min at a flow rate of 30 µl/min. Dissociation was investigated by injecting HBS-EP for 4 min at a flow rate 30 μl/min. Regeneration of the chip surface was established by injecting 3 M MgCl_2_ for 30 sec at a flow rate 30 μl/min. All kinetic parameters were calculated by nonlinear regression analysis according to a 1:2 binding model^47^, which reflects the avidity effect, using BIAcore T200 Evaluation Software (version 3.0). The dissociation constant, *K*_D_, was calculated using the formula *K*_*D*_ = *k*_*off*_*/k*_*on*_ (where *k*_*off*_ and *k*_*on*_ are the dissociation and association rate constants, respectively).

#### Nano-differential scanning fluorimetry (nanoDSF)

Thermal melting analysis of Abs was performed using a Tycho NT.6 instrument (NanoTemper Technologies). Ab samples were heated in a glass capillary using a linear thermal ramp (30 °C/min from 35 to 95 °C). The tryptophan fluorescence at 330 and 350 nm was recorded during heating, and all measurements were repeated three times. Data analysis and calculation of derivatives were performed using the internally automated evaluation features of the NT.6 instrument.

### Enzyme-linked immunosorbent assay (ELISA)

The 6C407 and 3D8 Ab series (1 μg/ml) in phosphate-buffered saline (PBS) were incubated in a 96-well polystyrene microtiter plate (ThermoFisher Scientific; cat# 439454) coated with 10 μg/ml HSET-N peptide or 5 μg/ml ss-(dN)_40_ antigen, respectively, for 1 h at room temperature. If necessary, Ab proteins were heated for 10 min to 4 h at 30–90 °C. Mouse IgG Abs bound to wells were detected using a rabbit anti-mouse IgG (Rockland; cat# 610-4503) followed by an alkaline phosphatase (AP)-conjugated goat anti-rabbit IgG (ThermoFisher Scientific; cat# 31341). Chimeric MH and MC Abs bound to wells were detected using rabbit anti-human IgG (ThermoFisher Scientific; cat# 31142) and rabbit anti-chicken IgY (Dianova; cat# 303-035-008) Abs, respectively, followed by an AP-conjugated goat anti-rabbit IgG. Color was developed by adding *p*-nitrophenyl phosphate substrate solution (1 mg/ml prepared in 0.1 M glycine, 1 mM ZnCl_2_, and 1 mM MgCl_2_, pH 10.3) to each well. The absorbance at 405 nm was read using a PowerWavex microplate reader (BioTek).

### Size-exclusion chromatography (SEC)

SEC analyses of purified Abs were performed using a DGU-20A3 UFLC system (Shimadzu) fitted with a TSK G3000SWXL column (7.8 × 300 mm; Toso Haas). Ab proteins were diluted with PBS to 1 mg/ml, and 30 μl of diluted Ab solution was injected onto the column. Where necessary, the protein was heated for 10 min, 2 h, or 4 h at 70 °C prior to injection. The mobile phase was 100 mM HEPES/85 mM HNaSO_4_ (pH 6.8), and the flow rate was 1 ml/min. Chromatograms were obtained by monitoring the absorbance at 280 nm.
